# Parliament and the Profession

**Published:** 1915-10-30

**Authors:** 


### PARLIAMENT AND THE PROFESSION.

### Home Hospital Reserves.

On October 19 Mr. Clough asked the Under-Secretary of State for War whether, under the present regulations, men of the Home Hospital Reserves are liable to serve either at Malta, Gibraltar, or elsewhere outside the United Kingdom. Mr. Tennant's reply was in the negative, but he pointed out that such men may volunteer for general service.

### The Health of the Troops in Gallipoli.

The Under-Secretary of State for War has given a provisional promise that he will shortly give particulars as to the number of troops now in hospitals in the Mediterranean area, and of those who have died from sickness in that area. In the meantime he has made the reassuring statement that in the case of dysentery, which has been the chief disease, the figures show a remarkable and gratifying decline since the beginning of this month.

### Hospital Stoppages.

On October 20 Mr. Tyson Wilson asked the Financial Secretary to the War Office if his attention had been called to the case of a private in a Yorkshire regiment, who, whilst in hospital, had 7d. per day stopped from his pay. Mr. Forster has promised to inquire into the matter, and in the meantime he understands that when a man goes to hospital the stoppages are deducted as a matter of course, until the medical certificate is received showing that the disease was not contracted through the soldier's own fault. This practice may prove hard on the soldier.

### Experiments on Living Animals.

Mr. Greenwood again referred to the question of the returns relating to experiments on living animals in the House of Commons on October 20 and 21. What he mainly desired to know was why the names of those licensed experimenters on living animals who have committed a breach of the law in the course of such experiments are suppressed. To this Sir John Simon made the reasonable reply that it had never been the practice to publish the names of persons guilty of technical offences or the omission of formalities which could be adequately dealt with by reprimand. Of course, if the offence were serious and a prosecution were instituted, the name would become public.

### Medical Help from Australia.

The misunderstanding with regard to the 10th Australian General Hospital, alluded to in The Hospital last week (p. 90), has been removed by an explanation furnished to the House of Commons by Mr. Tennant. It seems that the medical officers and nurses referred to do not constitute the complete staff of a general hospital. They arrived without equipment and were sent to this country in view of the contemplated extension of accommodation for Australians in the 3rd General Hospital, and to provide the staff for that purpose. Arrangements are in progress for such extensions, and it is proposed that the services of this staff shall be used in this connection.

### False Economy.

There is a danger that the public authorities may carry economy too far. A case in point was mentioned in the House of Commons on October 20, when Mr. Clancy asked the Vice-President of the Irish Department of Agriculture whether his attention had been directed to the resolution passed by the County of Dublin Insurance Committee relative to what is described as the great danger to the purity of the milk supply and the consequent results to the health of the community, especially of the young, caused by the withdrawal of the Bovine Tuberculosis Order. Mr. Birrell acknowledged that the resolution referred to was received by the department. He also admitted that all provisions of the Order sent out had been suspended in Ireland, as they had been in Great Britain, and for the same reason—namely, retrenchment of expenditure owing to the financial position arising from the war.

### A Solitary Panel Patient.

In the House of Commons on October 19 Mr. Currie asked the administrative head of the Insurance Act whether his attention had been drawn to the case of a panel doctor in Edinburgh who received £76 14s. 2d. for the solitary patient on his panel, who required no medical attendance whatever from one end of the year to the other. In his reply, Mr. C. Roberts stated that the remuneration of a doctor under the capitation system is governed by the terms of his agreement with the Insurance Committee, and includes not only his liability in respect of the persons actually on his list, but also the responsibility which he undertakes in common with other doctors on the panel for giving treatment to persons who have not selected a doctor for themselves. The payment made in the specific case referred to under the agreement entered into at the beginning of the year covered this liability. Mr. Currie returned to the matter on October 21, asking a series of questions which culminated as follows:—Mr. Currie: "Can the hon. gentleman say whether as a matter of fact this doctor did anything at all in exchange for the sum of £76, and is not such expenditure extravagant?" Mr. Roberts: "He held his services available for a considerable number of insured persons." And there the matter rests for the present.

### Drugs from Germany.

The difficulty of procuring certain classes of drugs in which Germany had held the monopoly up to the time of the outbreak of war has led to an official arrangement which, while it is hardly in accord with general policy, is nevertheless a wise one under the circumstances. The British Government, in fact, gives directions to His Majesty's ships that particular consignments to neutral countries are not to be captured and brought in for adjudication in a Prize Court. As regards goods ordered and delivered since March 1, 1915, such action has only been taken in the case of a few consignments of certain drugs unprocurable except from enemy territory or having no chemical equivalents procurable outside enemy territory, in the case of a few natural products, and certain scientific publications required by universities or educational establishments in a neutral country. These consignments have been allowed to pass either in response to urgent representations made by certain neutral Governments, based sometimes on undeniable humanitarian grounds, or to meet certain home demands. In making a statement on the matter in the House of Commons on October 21, Sir Edward Grey mentioned salvarsan and novocain as two of the drugs which are unprocurable except from enemy territory; as a matter of fact it is quite incorrect that the chemical equivalents of these drugs can only be obtained in an enemy country.

